# Insecticide Resistance Mechanisms in the Green Peach Aphid *Myzus persicae* (Hemiptera: Aphididae) II: Costs and Benefits

**DOI:** 10.1371/journal.pone.0036810

**Published:** 2012-06-07

**Authors:** Andrea X. Silva, Leonardo D. Bacigalupe, Manuela Luna-Rudloff, Christian C. Figueroa

**Affiliations:** Instituto de Ciencias Ambientales y Evolutivas, Facultad de Ciencias, Universidad Austral de Chile, Valdivia, Chile; U. Kentucky, United States of America

## Abstract

**Background:**

Among herbivorous insects that have exploited agro-ecosystems, the peach-potato aphid, *Myzus persicae*, is recognized as one of the most important agricultural pests worldwide. Uses over 400 plant species and has evolved different insecticides resistance mechanisms. As *M. persicae* feeds upon a huge diversity of hosts, it has been exposed to a wide variety of plant allelochemicals, which probably have promoted a wide range of detoxification systems.

**Methodology/Principal Findings:**

In this work we (i) evaluated whether insecticide resistance mutations (IRM) in *M. persicae* can give an advantage in terms of reproductive fitness when aphids face two hosts, pepper (*Capsicum annuum*) a suitable host and radish (*Raphanus sativus*) the unfavorable host and (ii) examined the transcriptional expression of six genes that are known to be up-regulated in response to insecticides. Our results show a significant interaction between host and IRM on the intrinsic rate of increase (*r_m_*). Susceptible genotypes (not carrying insensitivity mutations) had a higher *r_m_* on pepper, and the transcriptional levels of five genes increased on radish. The *r_m_* relationship was reversed on the unfavorable host; genotypes with multiple IRM exhibited higher *r_m_*, without altering the transcriptional levels of the studied genes. Genotypes with one IRM kept a similar *r_m_* on both hosts, but they increased the transcriptional levels of two genes.

**Conclusions/Significance:**

Although we have studied only nine genotypes, overall our results are in agreement with the general idea that allelochemical detoxification systems could constitute a pre-adaptation for the development of insecticide resistance. Genotypes carrying IRM exhibited a higher *r_m_* than susceptible genotypes on radish, the more unfavorable host. Susceptible genotypes should be able to tolerate the defended host by up-regulating some metabolic genes that are also responding to insecticides. Hence, our results suggest that the trade-off among resistance mechanisms might be quite complex, with a multiplicity of costs and benefits depending on the environment.

## Introduction

The evolution of insecticide resistance is one of the best-known examples of Darwinian microevolution on an ecological time-scale [1], [2]. In addition, given their negative impacts on crops and its economic consequences, the development of insecticide resistance in pest insects represents an important threat to human welfare [3]–[5]. Overall, insecticide resistance is based on several non-exclusive mechanisms: (i) behavioral evasion, (ii) thickening of the cuticle, (iii) increased activity of the metabolic machinery and, (iv) point mutations at insecticide target sites that reduce or eliminate insecticide sensitivity [3], [6]–[8].

Among the groups of herbivorous insects that have successfully exploited the agricultural environment, the green peach aphid, *Myzus persicae* (Sulzer), is recognized as one of the most important agricultural pests worldwide [9], [10]. This species uses over 400 plant species around the world from 50 different families [11], [12], and it causes damage both through direct feeding and by transmitting plant viruses. Although several insecticides have been used to control this species, *M. persicae* has developed resistance to all of them through either metabolic or target site mutation mechanisms [13], [14]. So far, four mechanisms of insecticide resistance through target site mutations have been described in this species: (i) modified AChE (MACE) [15]–[17], (ii) knock-down mutations (*kdr*) and super-*kdr* mutations in voltage-gated Na^+^ channels [18], [19], (iii) a mutation in the GABA-*Rdl* receptor [20], and (iv) the recently described mutation of a key residue in the loop D region of a nAChR b1 subunit [13]. Regarding the metabolic insecticide resistance, *M. persicae* shows resistance through over-production of E4 or EF4 esterases [21]–[26], and the recently reported over-production of cytochrome P450 [13], [27], [28].

Regarding those metabolic and target site insensitivity mechanisms, several authors have proposed that plant allelochemical detoxification systems found in insects have served as a pre-adaptation for the acquisition of insecticide resistance [8], [29]–[36].

Two lines of evidence have contributed to this hypothesis. First, insecticides not only resemble plant chemical defenses in their structure, but some are also derived from them(e.g. pyrethroids and neonicotinoids) [37], [38]. Second, the metabolic pathways involved in the detoxification of secondary metabolites in insects are highly conserved [39], [40]. Indeed, *M. persicae* feeds on a wide diversity of hosts, therefore being exposed to a range of phytochemicals, thus favoring a great diversity of enzymatic detoxification systems [41]–[43]. Detoxification mechanisms against allelochemicals, however, are poorly investigated in *M. persicae* [44]. Nevertheless, it has recently been reported that esterases play a role in the ability of the tobacco aphid (*Myzus persicae nicotianae*) to feed on tobacco plants (*Nicotiana tabacum*) [45], while glutathione S-transferases participate in detoxifying glucosinolates and isothiocyanates characteristic of the Brassicaceae family [46].

The fecundity of adult aphids is one of the most frequently studied traits used to characterize the ability to feed on different hosts, because it is expected that specialization in herbivorous insects has evolved towards an optimal exploitation of the host in terms of maximizing individual fitness [47]. In this work, we explored the reproductive and transcriptional responses of *M. persicae* clones carrying different insecticide resistance mechanisms under distinct environmental regimes imposed by the host plant. First, we evaluated the reproductive fitness of different aphid genotypes carrying or not MACE and *kdr* insensitivity mutations, which were reared on suitable and unfavorable host plants. Second, we compared the transcriptional levels for six specific genes on aphid genotypes reared on both hosts. In particular, we selected some genes coding for Cathepsin B, Heat Shock Protein 70, Glutathione S-Transferase, Carboxylesterase and Cytochrome p450 family CYP6 and CYP4. We selected those genes because they showed the highest up-regulation in expression (ranging 2–5 fold change) in a previous work where *M. persicae* individuals were subjected to insecticides [48].

## Results

### Insecticide Resistance Assessment

Of a total of 44 multilocus genotypes studied, thirty-three did not carry any resistance mutations and were labeled as sensitive (i.e. S). Six genotypes were heterozygote for *kdr* mutation and five were heterozygote for both *kdr* and MACE mutations. These genotypes were labeled simple resistant (i.e. SR) and multiple resistant (i.e. MR), respectively. No genotypes was found to carry either MACE or *kdr* mutations in homozygote state, or carrying a super-*kdr* mutation. Of the 44 genotypes evaluated for constitutive carboxylesterase activity (EST activity), thirty-two genotypes were classified as susceptible (S), ten as moderately resistant (R1) and two were highly resistant (R2), following the nomenclature proposed by Devonshire et al. (1992) [49]. No genotype was found to be extremely resistant (R3). Nine genotypes (N36-1, Teno7B, Sur25A, 26A, N30A-1, Cruz 4A, Peralillo 1, Sur 74-1 and 16A) were selected for the experiments, considering different genetic configuration for insecticide resistance mutations (IRM) and EST activity (Table 1).

**Table 1:** **Genetic configuration for insecticide resistance mutation (IRM) and constitutive esterase activity (EST)of selected genotypes.**

Genotype	IRM			Genotype IRM	EST activity	Genotype EST
	MACE	*kdr*	*s.kdr*		(U aphid-equiv. ^−1^)	
**N 36-1**	**SS**	**SS**	**SS**	**S**	**0.133±0.05**	**S**
Teno7B	SS	SS	SS	S	0.091±0.01	S
**Sur 25A**	**SS**	**SS**	**SS**	**S**	**0.300±0.02**	**S/R1**
26A	SS	SR	SS	RS	0.207±0,01	S
N 30A-1	SS	SR	SS	RS	0.390±0.06	S/R1
Cruz 4A	SS	SR	SS	RS	0.359±0.09	S/R1
**Peralillo 1**	**SS**	**SR**	**SS**	**RS**	**0.701±0.08**	**R1/R2**
Sur74-1	SR	SR	SS	RM	0.142±0.02	S
**16A**	**SR**	**SR**	**SS**	**RM**	**0.291±0.05**	**S**

IRM genotypes were assigned according to whether or not the insects carried the IRM being studied. Thus, genotypes that did not carry any resistance mutations and were labeled as sensitive (S), genotypes were heterozygote for *kdr* were labeled as simple resistant (SR) and heterozygotes for both *kdr* and MACE mutations were labeled as multiple resistant (MR). Genotypes for EST activity were assigned following the nomenclature proposed by Devonshire et al. (1986, 1992). Thus, genotypes were classified as susceptible (S), moderately resistant (R1) and highly resistant (R2). Values are means ± SE. The genotypes shown with dark background were used for RT-qPCR experiments.

### Reproductive Fitness

**Figure 1:**
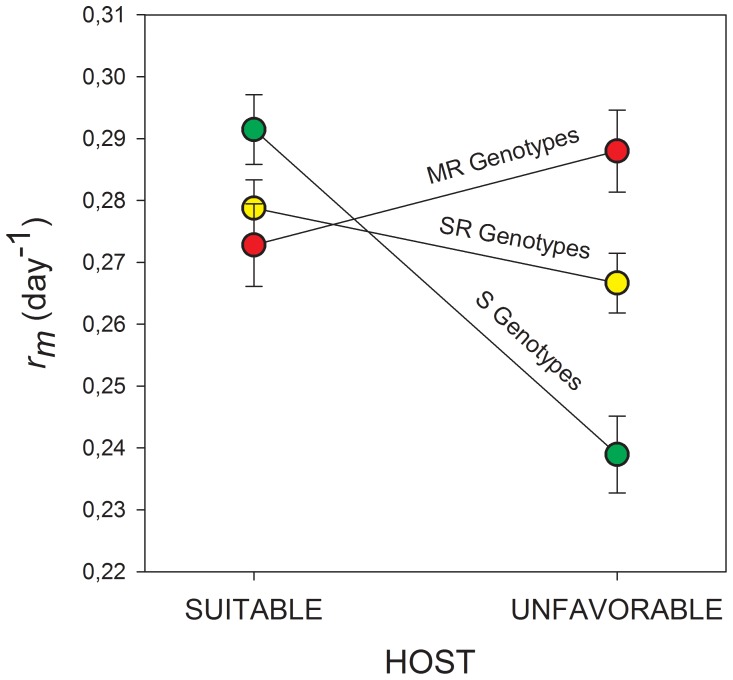
Norms of reaction in reproductive fitness (*r_m_*) of *Myzus persicae* genotypes in different host. For nine genotypes with three different genetic configurations of insecticide resistance mutations (IRM) is shows the mean in *r_m_* (mean ± SE) in two host, pepper (suitable) and radish (unfavorable).The green circles correspond to mean in genotypes sensitive (S, N = 3), the yellow circles corresponds to mean in simple resistant (SR, N = 4), and the red circles corresponds to multiple resistant genotype (MR, N = 2). The interaction HOST X IRM was significant (*F*_2,6_ = 5.771, P = 0.040, from nested ANOVA).

Table 2 shows descriptive statistics for intrinsic rate of increase (*r_m_*) and body mass, for each tested genotype on both host plants (suitable and unfavorable). The results shown no variation for reproductive fitness (χ^2^ [_1_] = 0.000001, P = 0.999) among lines within genotypes; thus, line was removed from the final model. On the other hand, reproductive fitness was, as expected, positively affected by body mass (*b* = 0.227, SE = 0.020; *F*_1, 230_ = 131.42, P<0.0001). In addition, not all genotypes responded in the same fashion to both hosts; that is, there was an interaction between genotype and host (χ^2^ [_1_] = 8.477, P = 0.004). Within each genotype, aphid reproductive fitness was significantly lower on the unfavorable host (radish) for S genotypes (N36-1, Sur25A). Finally, our results also shown a significant interaction between host and IRM on reproductive fitness (*F*_2,6_ = 5.771, P = 0.040). In particular, on pepper, the S genotypes had a higher reproductive fitness than SR and MR genotypes. However, the relationship was reversed on radish: S genotypes showed a lower reproductive fitness than SR and MR ones (Figure 1).

**Table 2:** **Descriptive statistics for intrinsic rate of increase (** ***r_m_*** **) and body mass for each tested genotype.**

Genotype	Pepper (suitable host)			Radish (unfavorable host)		
	*r_m_*(day^-1^)	mass (µg)	N	*r_m_* (day^-1^)	mass (µg)	N
**N 36-1**	**0.30±0.01**	**0.48±0.04**	**15**	**0.23±0.02**	**0.48±0.04**	**8**
Teno7B	0.29±0.01	0.36±0.03	16	0.28±0.01	0.42±0.03	15
**Sur 25-A**	**0.31±0.01**	**0.51±0.04**	**8**	**0.22±0.02**	**0.46±0.04**	**9**
26A	0.21±0.01	0.28±0.03	14	0.24±0.01	0.49±0.03	15
N 30A-1	0.28±0.01	0.35±0.03	15	0.31±0.01	0.52±0.02	10
Cruz 4A	0.27±0.01	0.38±0.02	16	0.32±0.01	0.53±0.02	16
**Peralillo 1**	**0.30±0.01**	**0.44±0.03**	**16**	**0.26±0.01**	**0.41±0.03**	**14**
Sur74-1	0.26±0.01	0.34±0.03	14	0.30±0.01	0.49±0.03	12
**16A**	**0.26±0.01**	**0.42±0.04**	**13**	**0.20±0.01**	**0.40±0.03**	**15**

Shown values of *r_m_* when aphids were reared on pepper (*Capsicun annuum* var. *grossum*) and radish (*Raphanus sativus* var. *sparkler*), followed by body mass and sample size (N). Values are means ± SE. The genotypes shown with dark background were used for RT-qPCR experiments.

### Transcriptional Levels of Candidate Genes

**Figure 2:**
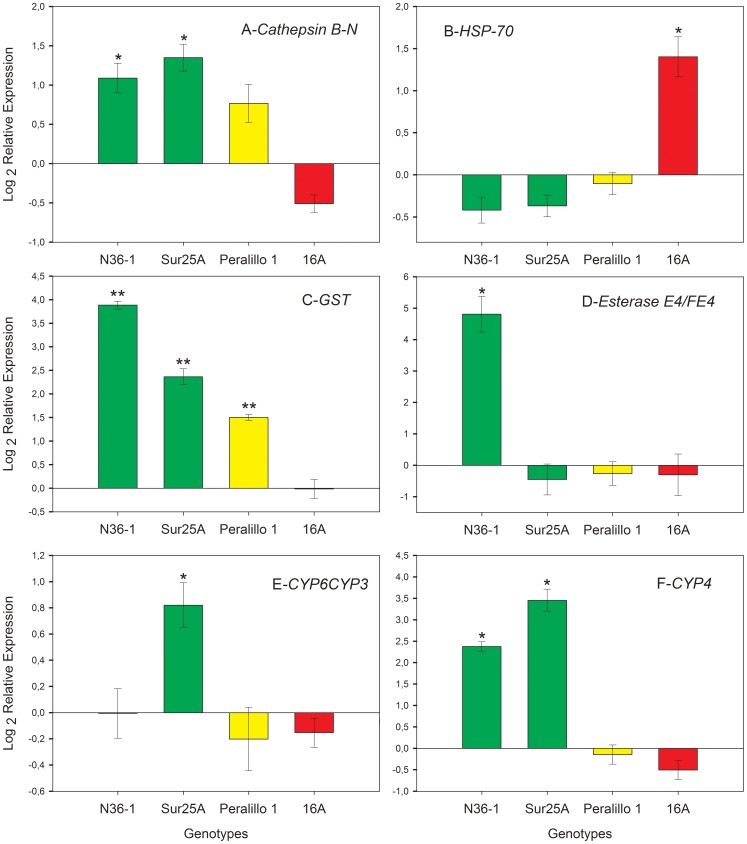
Quantification of relative expression levels in four genotypes on suitable (pepper) and unfavorable (radish) hosts. The results represent the relative mRNA expression, with transcripts expressed by the aphids on pepper as calibrator and on radish as interest sample. Data were normalized for variation using GADPH expression. The green bars correspond to S genotypes (sensitive; N36-1 and Sur25A), the yellow bar corresponds to the SR genotype (simple resistant; Peralillo 1) and the red bar corresponds to the MR genotype (multiple resistant; 16A). Data represent mean ± SE of two different experiments, with three technical replicates each case.*p<0.05 and **p<0.01 indicate a significant difference compared to 1, used as a reference value for no change in expression using a *t-test*. Gene abbreviations: (A) *cathepsin B-N*, *cathepsin B clade N*; (B) *HSP-70*, *heat shock protein 70*; (C) *GST*, *glutathione S-transferase*; (D) *Esterase E4/FE4*, *carboxylesterase type E or FE4*; (E) *CYP6CY3*, *cytochrome p450family CYP6CYP3*; (F) *CYP4*, *cytochrome p450 family CYP4*.

The transcriptional level for all six of the selected genes depended on the genotype (Figure 2).

*Cathepsin B gene* – This gene showed a significant up-regulation only in the S genotypes (N36-1 and Sur25A) when aphids were reared on radish (Figure 2A). Genotypes SR (Peralillo 1) and MR (16A) showed only a slight but not significant up and down-regulation, respectively.

*Heat Shock Protein 70 gene –* A significant up-regulation was observed only in the genotype MR (16A) when reared on radish. The transcriptional levels for the remaining genotypes was not significantly different from 1 (Figure 2B).

*Glutathione-S-transferase gene* – The relative expression of the *GST* gene was significantly higher for three aphid genotypes reared on radish compared to aphids reared on pepper; only the MR genotype showed no significant differences in relative expression between hosts (Figure 2C).

*Esterase gene* – Only one genotype (N36-1) showed an up-regulation of the *E4/FE4* gene when aphids were reared on radish (Figure 2D). The transcriptional levels for the *E4/FE4* gene were not significantly different from 1 in the other genotypes.

*Cytochrome P450 genes* – Two genes belonging to this family were assessed (*CYP6CY3* and *CYP4*). The genotypes SR and MR evidenced a slight but not significant down-regulation for both genes (Figure 2E and 2F). However, both S genotypes showed an up-regulation for the *CYP4* gene (Figure 2F), while only the S genotype Sur 25A showed a higher relative expression for the *CYP6CY3* gene (Figure 2E) after rearing aphids on radish.

## Discussion

In this work we examined the potential involvement of specific genes to determine whether point mutations at insecticide target sites (MACE and *kdr*) found in a pest aphid in Chilean agroecosystems can provide an advantage in terms of reproduction success when aphids are faced with well-defended host plants. This is particularly interesting when studying a highly polyphagous insect that is able to feed on more than 50 different plant families exhibiting a vast range of chemical defenses against herbivorous. The subject of this study was to determine whether the diversity of possible hosts and their defenses has promoted the evolution of different aphid counter-defense mechanisms that can be also involved in insecticide resistance. Therefore, we evaluated the impact of host plants with different levels of allelochemicals on: (i) the reproductive performance of aphid genotypes carrying or not MACE and *kdr* insensitivity mutations and, (ii) the transcriptional expression for six selected genes. Particularly, we selected genes coding for Cathepsin B, Heat Shock Protein 70, Glutathione S-Transferase, Carboxylesterase and Cytochrome p450 family CYP6 and CYP4, as they were observed to be highly up-regulated (ranging 2–5 fold change) in a previous work where *M. persicae* genotypes were subjected to insecticides [48].

Our results highlight a number of aspects regarding the complexity of the relationships between phytophagous insects and their host plants in heterogeneous environments. Clearly, genotype-specific traits and genotype-by-environment interaction are determinant of aphid’s gene expression pattern and overall performance. Thus, it is neither straightforward nor simple to make generalizations. In spite of this, a few common features can be identified.

### Inter-clonal Variation and Potentiality to Adaptive Evolution

The fitness among genotypes exhibited a high variation on the two tested hosts (pepper or suitable host and radish or unfavorable host). This significant inter-clonal variation in performance has also been reported by other authors [50]–[53], suggesting that populations of *M. persicae* have the potential to evolve in response to selection agents like host plants.

### Environmental Canalization versus Phenotypic Plasticity

Although we have only studied nine genotypes, in overall terms our results are in agreement with the general idea that allelochemical detoxification systems can serve as a pre-adaptation for the development of insecticide resistance. In our case, the SR and MR genotypes exhibited a higher reproductive fitness than S genotypes on the defended host. However, costs and benefits associated with phenotypic expression depend on the locations of fitness optima in different environments [54], [55]. Thus, individuals that carry insensitivity mutations or constitutive overproduction of detoxification enzymes could have higher costs in environments without insecticides or plant chemical defenses compared to undefended individuals. It is known that insecticide insensitivity mutations are associated with fitness costs [56], [57]. Pleiotropic effects of E4/FE4 gene duplication and *kdr* mutation on important behavioral traits have been reported for *M. persicae*, particularly in response to environmental signals such as presence of natural enemies (parasitoids) and low temperatures, which ultimately could lead to reduced fitness [58]–[63]. In addition, the MACE mutation appears to reduce the fitness of individuals that carry it [64]. However, the effect of this mutation by itself has not been clarified yet [65], [66]. This might explain the inverse relationship we observed in aphids reared on pepper: genotypes SR and MR having a lower reproductive fitness than S genotypes. However, SR and MR genotypes did not change significantly their reproductive fitness between hosts, and present a weak transcriptomic response, suggesting an environmental canalization.

On the other hand, the overall lower reproductive fitness showed by S genotypes on radish might be indicative of a cost. Two S genotypes (N36-1 and Sur25A) showed the greatest variation in fitness between hosts and thus were selected for transcriptional analyses. Those S genotypes were also the most plastic in terms of transcriptional variation, showing an important up-regulation in five of the six genes evaluated on the defended host. Therefore, the lower reproductive fitness shown by these two genotypes on the defended host might be indicative of an energetic trade-off, suggesting non-adaptive plasticity [67]–[69]. In fact, it has been reported that metabolic detoxification mechanisms in insects are energetically expensive, which would result in an allocation trade-off between defense mechanisms and other biological functions such as growth and reproduction [70]–[72]. Certainly, this trade-off allows aphids to survive on the defended host, but at a lower reproductive rate.

### Transcriptional Plasticity and the Evolution of Pre-adaptation

If resistance to plant chemical defenses is a *pre-adaptation* that enables aphids to deal with insecticides, then it would be expected that genotypes carrying no resistance mechanism should be able to tolerate the defended host by up-regulating specific metabolic genes in the same way as they do when exposed to insecticides [48]. Overall, this is what we found for most of the studied genes (*cathepsin B clado N*; *heat shock protein 70*; *glutathione S-transferase*; *Esterase E4/FE4*, *cytochrome p450* family *CYP6CYP3* and *CYP4*).

The Cathepsins B are enzymes with cysteine protease activity that process exogenous polypeptides into aminoacids, which are used to synthesize their own proteins by sap-sucking insects [73]–[75].Also, these proteases are up-regulated to minimize the effects of plant protease inhibitors (PIs) in some insects[76]–[79]. In aphids, including *M. persicae*, PIs interfere with aminoacid assimilation, having a negative impact on fitness [74], [80]. In addition, these defense mechanisms have been reported in Brassica plants [74], [81], [82]. Our results show a significant up-regulation of the *cathepsin B* gene in both S genotypes. This could be a counter-defense mechanism against the PIs in radish plant or to supplying aminoacids for the biosynthesis of the enzymatic machinery needed for detoxification.

Heat shock proteins-70 (Hsp70) are a family of well known proteins involved in cell protection and repair, reducing protein aggregation and unfolding no-native protein conformations caused by environmental stress [83]–[86]. The up-regulation of Hsp70 genes has been reported in insects exposed to pesticides [87]–[89], thermal stress [89]–[91], oxidative stress [92], and metals [89], [93], among other stressors. In our study, the genotype MR was the only one showing a significant Hsp70 up-regulation after being reared on radish. Furthermore, this was the only gene that was significantly over-expressed by this genotype. On the other hand, the transcriptional expression for the remaining genotypes was not different from 1, probably because other detoxifying mechanisms are preventing the level of stress from reaching a threshold level.

The other studied genes belong to the three gene families that typically participate in detoxification of plant chemical defenses and metabolic resistance to insecticides: *glutathione S-transferases* (GST), *cytochrome P450* (P450s proteins, encoded by *CYP* genes) and *carboxylesterases* (ESTs).GST enzymes participate in the detoxification of xenobiotic substrates by conjugation of glutathione to electrophilic toxic molecules [30], [94]. Increments in the activity of GST enzymes in *M. persicae* have been reported as a response to glucosinolates and isothiocyanates [46], which are characteristic of Brassicaceae plants such as radish [11], [95]–[99]. Thus, it is not unexpected that the relative expression of the *GST* gene was consistently higher in S and RS genotypes reared on radish.

ESTs participate in the sequestration and hydrolysis of esters and amides [8], [21], [100]. Metabolic insecticide resistance (to organophosphates, pyrethroids and carbamates) through increased ESTs activity is one of the several mechanisms reported in *M. persicae*. However, this metabolic resistance is due to up to 80-fold duplication of the carboxylesterases genes (*E4* and *FE4*) [101], [102]. We found that the S genotype (N36-1) was the only one showing a 30-fold up-regulation of the *E4/FE4* gene when aphids were reared on the defended host. The other S genotype studied (South 25 A), had a constitutive E4/FE4 expression more than twenty-fold than genotype N36-1 (expression among genotypes reared on the most suitable host; data not shown), which could explain the absence of up-regulation of this gene in that genotype.

P450 enzymes oxidize a broad range of endogenous and exogenous lipophilic compounds [8], [40], [103]–[105]. More than 660 *CYP* genes have been characterized in several insect orders [8], with the *CYP6*, *CYP3* and *CYP4* families being the most important in detoxifying plant defenses and insecticides [105]. For most of the tested genes, transcriptional levels were dependant on the genotype. Particularly, the S genotypes showed higher levels of expression when aphids were reared on the defended host.

In general, for the six genes studied, the low transcriptional plasticity exhibited by aphid genotypes SR and MR suggest a greater constitutive expression of these or other related genes, including paralogs for those studied here. While the SR genotype up-regulated only one detoxifying gene (*GST*), it showed a constitutively high level of EST activity (Table 1). In fact, the *E4/FE4* expression among genotypes reared on the suitable host showed that SR genotype presented fifty times more transcripts in average (data not show) than the other genotypes. In the case of the MR genotype, only the *hsp-70* gene was up-regulated, but constitutively showed approximately three times more transcripts for *GST* than in the other genotypes (data not shown). This kind of canalization in response to defended plants has been recently reported for the grain aphid *Sitobion avenae*. Using the so-called ‘superclones’ (i.e. the most common and time-persistent genotypes) of the grain aphid reared on highly defended cereals, aphids demonstrated a rigid detoxifying capacity. That is, they did not modify detoxification enzyme activities with a similar cost across defended and non-defended plants [106]. It has been suggested that detoxification systems demand fitness and metabolic costs only when aphids are reared on poorly defended plants [107]. On the other hand, a transcriptomic study in *M. persicae* at the whole genome level showed that in general the S genotype avoided the lethal effects of insecticide by up-regulating 183 genes [48]. In contrast, that study also showed the SR and MR genotypes up-regulated only 17 and 7 genes respectively, with the most insensitive genotype showing rigidity for the expression of genes encoding enzymes involved in insecticide detoxification. Therefore, the lower reproductive fitness observed in genotypes carrying insensitivity mutations, in comparison with sensitive genotypes on the susceptible host, could be explained in terms of the inability to turn off the detoxification machinery.

In conclusion, our results suggest that aphids under “insecticide” or “chemically defended plants” conditions have similar adaptive solutions to two different selective agents. Although we have only studied nine genotypes, their overall response in both reproductive performance and transcriptional expression was fairly consistent across genotypes carrying or not carrying MACE and *kdr* insensitivity mutations reared on suitable and unfavorable hosts. Thus, our results suggest that the trade-off among resistance mechanisms (by detoxification or insensitivity) might be quite complex, with a multiplicity of costs and benefits between environments. All of the selective agents (i.e. predators, climate, temperature, plant allelochemicals, and insecticides) play key roles in shaping population structures. Studies that consider the spatial and temporal dynamics of aphids are needed to understand the cost/benefit balance of the mechanisms herein studied. Finally, more research is certainly needed to confirm the generality of our findings and to determine and understand the wide arrange of phenotypic correlates of detoxification responses that *M. persicae* can exhibits.

## Materials and Methods

### Aphid Genotypes

Ninety four clonal lineages (genotypes) previously sampled and established in the laboratory were used in this study and genotyped using six microsatellite loci (for details see Castañeda et al. 2011) [108]. Among these, 44 genotypes were selected for study their insecticide resistance mechanisms, from these nine were selected for reproductive fitness experiments and finally four of these were used to quantitative reverse transcription PCR experiments. Each aphid genotype was reared on seedlings of pepper (*Capsicum annuum* var. *grossum*) in Blackman boxes under conditions that ensure parthenogenetic reproduction (20±1°C and L: D 16∶8). Colonies were maintained by transferring 5 wingless adults on new 7-day-old pepper seedlings every 10 days for at least 20 generations before the experiments.

### Insecticide Resistance Assessment

The presence of insecticide resistance mutations (IRM) was screened in the 44 genotypes, using allelic discrimination based on quantitative-PCR assays developed by Anstead et al. (2004) for *kdr* (L1014F) and super*-kdr* (M918T) mutations, and by Anstead et al, (2008) for the MACE mutation [109], [110].Constitutive carboxylesterase activity (EST activity),indicative of the genotype respect to the number of copies for E4/FE4 carboxyl esterase genes [101], [102], was evaluated in the same genotypes using the microplate bioassay [49], [111], with five independent biological replicates and three technical replicates per measurement.

### Breeding Design and Reproductive Fitness Determination

The host-plant effect on the fitness of *M. persicae* was conducted by estimating the intrinsic rate of natural increase (*r_m_*) when aphids were reared on pepper (*C. annuum*var. *grossum*), the most suitable host for *M. persicae* [112], and radish (*Raphanus sativus* var. *sparkler*), an unfavorable host species [53] in which glucosinolates (GLS) have been described as the main defense systems against aphids [41], [95].

One single adult wingless aphid (parental) from each selected genotype (Table 1) was transferred to a nine 3-months-old sweet pepper or radish plant and left to reproduce for 24 to 48 hours. Two parthenogenetic nymphs were maintained on the same plant until adulthood, discarding the rest of the aphids, giving rise to two lines per genotype and host. Each of these aphids was then transferred to a new 3-month-old plant and maternal and grand maternal effects were erased by 3 rounds of parthenogenetic reproduction on sweet pepper and radish. At the end of this procedure, 8 individual sub-lines for each line by genotype and host were obtained.

The intrinsic rate of natural increase (*r_m_*) for each genotype on each host was estimated following Wyatt and White (1977). The days from birth to first reproduction (*T*_d_) and the number of offspring produced in that time (*M*_d_) were determined. Then, the intrinsic rate of natural increase was calculated as *r_m_* = 0,738 (log_e_ *M*_d_)/*T*_d_, where 0,738 is a correction factor [113]. Aphids were cooled in ice for a few seconds and weighed to the nearest microgram on a microbalance MXA 5/1 (Radwag, Czech Republic).

### Quantitative Reverse Transcription PCR (RT-qPCR)

Transcriptional levels of six genes (*cathepsin B clado N*; *heat shock protein 70*; *glutathione S-transferase*; *esterase E4/FE4*, *carboxylesterase tipo E or FE4*; *cytochrome* p450 family CYP6, CYP3 and CYP4) that are known to be regulated when S and SR aphid genotypes for IRM are exposed to carbamate insecticides [48] were evaluated using RT-qPCR. Two sub-lines (randomly selected) per host in four genotypes (see genotypes shown with dark background in Table 1) were used for RT-qPCR experiments. After estimating *r_m_*, at least 3 nymphs from each line were maintained on its host plant until they were up to 12 days old. Aphids were collected from their host plants and immediately frozen in liquid N_2_ until RNA extraction.

Total RNA was isolated using the RNeasy Plant Mini Kit (Qiagen, Cat no. 74904) from three aphids per genotype and condition. From approximately 1.5 µg total RNA (previously treated with DNA-free™ kit Ambion), cDNA synthesis was conducted using AffinityScript QPCR cDNA Synthesis kit (Agilent). Then, the cDNA was diluted to 1∶10, taking 2 µl for PCR reactions (25 µl final volume). Each PCR reaction mix contained 10 pmol of each primer, 12.25 µl SYBR Green PCR Master Mix (Applied Biosystems) and 0.375 µl of Rox (dilution 1∶500), the latter used as passive reference dye. Negative controls were included for detecting foreign contamination, and all PCR reactions were performed in triplicate in a Mx3000P QPCR Systems (Stratagene) under the following cycling conditions: 10 min at 95°C, followed by 40 cycles of 15 s at 95°C, 15 s at 57°C and 20 s at 72°C. A dissociation curve was included immediately after each PCR using a ramp of 65–95°C, to confirm the absence of nonspecific amplifications. Primers were designed from the sequences of *M. persicae* contigs for six target genes (EC387286, EE261252, EC387215, EE262012, EC388935, EE263097) and one endogenous control gene (DW011095), using the FastPCR (V 5.4.30) and AmplifX (V 1.3.7) packages, and checked in NCBI/Primer-BLAST (for details in primer sequences and PCR efficiencies see Silva et al. 2012) [48].

### Statistical Analysis

We used a linear mixed modeling approach to evaluate the effect of IRM and host on *r_m_*, taking into account the presence of random factors (genotype, and genotype * host interaction), the nested structure of our design (i.e. the presence of mutations were genotype-specific, clonal lines were nested into genotypes) and some minor unbalances. Body mass was included as a covariate. Hypothesis testing for fixed effects was based on marginal *F* tests and for random effects was based on likelihood ratio tests of nested models [53]. Statistical analyses were performed using the NLME package [114] implemented in R platform 2.10.1 (R Development Core Team, 2009).

The relative expression ratio of a target gene was computed by relative quantification using the comparative Ct method (Applied Biosystems User Bulletin No. 2 P/N 4303859, 1997) [115], with the *glyceraldehyde-3-phosphate dehydrogenase* (GADPH) gene as normalizing endogenous control. Several studies have validated the use of GAPDH as a reference gene for normalization [116]–[118]. Furthermore, this gene has been shown to be one of the most stable endogenous genes in response to insecticides in the aphid *M. persicae* (fold change range 0.94 – 0.99) [48]. Ratios were calculated from a mean normalized expression (MNE) value obtained between biological replicates, as they show the same trend in all cases, with MNE values obtained from aphids maintained in sweet pepper as a calibrator. In each case we performed a t-test between the average and 1, which was considered as the reference value for no change in relative expression.
